# GnRH-1 Neural Migration From the Nose to the Brain Is Independent From Slit2, Robo3 and NELL2 Signaling

**DOI:** 10.3389/fncel.2019.00070

**Published:** 2019-03-01

**Authors:** Ed Zandro M. Taroc, Jennifer M. Lin, Alastair J. Tulloch, Alexander Jaworski, Paolo E. Forni

**Affiliations:** ^1^Department of Biological Sciences, University at Albany, Albany, NY, United States; ^2^Department of Neuroscience, Brown University, Providence, RI, United States

**Keywords:** GnRH-1, olfactory bulbs, Kallmann syndrome, hypogonadotropic hypogonadism, neuronal migration, Robo, Slit, NELL2

## Abstract

Gonadotropin releasing hormone-1 (GnRH-1) neurons play a pivotal role in controlling pubertal onset and fertility once they reach their hypothalamic location. During embryonic development, GnRH-1 neurons migrate from the nasal area to the hypothalamus where they modulate gonadotropin release from the pituitary gland. Defective migration of the GnRH-1 neurons to the brain, lack of GnRH-1 secretion or signaling cause hypogonadotropic hypogonadism (HH), a pathology characterized by delayed or absence of puberty. Binding of the guidance cue Slit2 to the receptor roundabout 3 (Robo3) has been proposed to modulate GnRH-1 cell motility and basal forebrain (bFB) access during migration. However, evidence suggests that Neural EGFL Like 2 (NELL2), not Slit2, binds to Robo3. To resolve this discrepancy, we analyzed GnRH-1 neuronal migration in NELL2, Robo3, and Slit2 knock-out mouse lines. Our data do not confirm a negative effect for monogenic Robo3 and Slit2 mutations on GnRH-1 neuronal migration from the nasal area to the brain. Moreover, we found no changes in GnRH-1 neuronal migration in the brain after NELL2 loss-of-function. However, we found that Slit2 loss-of-function alters the patterning of GnRH-1 cells in the brain, suggesting that Slit2 loss-of-function affects GnRH-1 cell positioning in the brain in a Robo3 independent fashion. Our results challenge previous theories on GnRH-1 neuronal migration mechanisms and provide a new impetus to identify and understand the complex genetic mechanisms causing disorders like Kallmann syndrome (KS) and HH.

## Introduction

Precise control of neural migration and circuit formation is essential to coordinate animal behavior and neuroendocrine functions. In mammals, pubertal onset and fertility requires correct development of gonadotropin releasing hormone-1 neurons (GnRH-1ns). During early embryonic development, these neurons migrate from the nasal area to the brain (Schwanzel-Fukuda and Pfaff, 1989). Once GnRH-1ns reach the hypothalamus, they secrete GnRH-1 peptide to control the release of follicle-stimulating hormone and luteinizing hormone from the pituitary gland (Forni and Wray, 2015). Lack of GnRH-1 secretion or signaling causes hypogonadotropic hypogonadism (HH), a pathology characterized by delayed or absent pubertal onset and sterility. Kallmann syndrome (KS; Dodé and Hardelin, 2009) is a form of HH associated with defective olfactory development resulting in anosmia (partial or total inability to smell).

The genesis of GnRH-1ns in the olfactory pit, and their migration from nose to hypothalamus are complex developmental processes that involve paracrine signals (Hardelin and Dodé, 2008; Trarbach et al., 2010; Forni et al., 2013; Zhang et al., 2015), migratory signals, axonal guidance cues (Toba et al., 2008; Cariboni et al., 2011, 2015; Messina et al., 2011; Casoni et al., 2012), as well as the interaction between cells types of different embryonic origins (Barraud et al., 2013; Forni et al., 2013; Pingault et al., 2013). Though large genetic screenings have led to the identification of a number of genes associated with KS and HH (Boehm et al., 2015; for an updated catalog visit http://OMIM.org), many still remain unknown. Moreover, a growing number of reports refute the long-existing view that KS and HH are monogenic diseases, proposing, instead, a more complex oligogenic nature for these genetic pathologies (Sykiotis et al., 2010; Balasubramanian et al., 2014; Stamou and Georgopoulos, 2018).

Diffusible and/or juxtacrine cues interact with receptors expressed on neuronal growth cones, define axonal trajectories, regulate neuronal migration, and produce accurate circuit formation. Two receptors, roundabout (Robo)1 and Robo2, cause axonal repulsion in response to Slit proteins, whereas a different Robo protein, Robo3, can silence Robo1 and 2-mediated Slit repulsion (Marillat et al., 2004; Sabatier et al., 2004; Chen et al., 2008; Jaworski et al., 2015) without binding to Slits (Zelina et al., 2014; Jaworski et al., 2015). Robo3 also increases deleted in colorectal cancer (DCC) mediated sensitivity to Netrin attraction and mediates axonal repulsion to its recently discovered ligand Neural EGFL Like 2 (NELL2; Zelina et al., 2014; Jaworski et al., 2015). The developing forebrain of mice is a source of Slit proteins (Nguyen-Ba-Charvet et al., 2002, 2004, 2008) which are expressed in non-overlapping fashion (Andrews et al., 2007). Slit1 and Slit2 released from the forebrain act as repellants preventing olfactory and vomeronasal neurons, which express Robo1 and/or Robo2, from invading the forebrain (Taroc et al., 2017). However, the terminal nerve (TN), upon which GnRH-1ns migrate to the brain, invades the brain and crosses key areas of Slit production (Schwanzel-Fukuda and Pfaff, 1989; Taroc et al., 2017).

Mice lacking the Slit receptors Robo1 and Robo2 can have severe defects in the development of the olfactory system (Nguyen-Ba-Charvet et al., 2008), but show no defects in GnRH-1ns migration or patterning (Cariboni et al., 2012), which suggests a lack of central control for the two main receptors for Slits in guiding GnRH-1 cells. However, Robo3^null^ and Slit2^null^ animals have been reported to have severe GnRH-1 neuronal migratory defects with approximately a 50% reduction in GnRH-1ns accessing the brain as they accumulate in the nasal area (Cariboni et al., 2012). Based on these data, a Robo3-Slit2 ligand-receptor axis has been proposed to guide the GnRH-1ns (Cariboni et al., 2012). Conversely, it is known that direct Slit2-Robo3 binding does not occur (Camurri et al., 2005; Zelina et al., 2014; Jaworski et al., 2015), which leaves the mechanism responsible for GnRH-1 neuronal migration in Robo3^null^ and Slit2^null^ mutants unresolved and controversial (Cariboni et al., 2012).

GnRH-1ns migrate from the nasal area to the brain along the projections of the TN (Schwanzel-Fukuda and Pfaff, 1989; Wray et al., 1989a,b). The migration of GnRH-1ns in Arx-1 null mutants, a mouse line with severe defects in olfactory bulb (OB) development, indicated GnRH-1ns can successfully migrate to the forebrain even without a proper olfactory connection to the brain (Taroc et al., 2017). Compared to TN and GnRH-1ns, these results also suggested that olfactory and vomeronasal neurons may express different Robo receptors and show different sensitivity to guidance cues in the Slit family (Taroc et al., 2017). TN and GnRH-1ns can migrate across sources of Slit proteins and express Robo3 (Cariboni et al., 2012; Taroc et al., 2017), which could make these neurons insensitive to Slit mediated repulsion by silencing Robo1 and Robo2 (Sabatier et al., 2004; Camurri et al., 2005; Chen et al., 2008; Zelina et al., 2014; Friocourt and Chédotal, 2017). We hypothesized that GnRH-1ns migratory defects previously reported for Robo3 and Slit2 mutants (Cariboni et al., 2012) could arise either from aberrant TN and GnRH-1ns responses to NELL2 mediated repulsion (Jaworski et al., 2015) or a loss in competency to switch off Slit mediated repulsion in a Robo3 dependent fashion (Sabatier et al., 2004).

## Materials and Methods

### Animals

All mice used in this study are on a CD-1 background of either sex. Generation and validation of Robo3, NELL2 and Slit2 null mutants were previously described (Plump et al., 2002; Sabatier et al., 2004; Jaworski et al., 2015). All animal procedures were approved by University at Albany Animal Care and Use Committee (IACUC).

### Tissue Preparation

Embryos were collected from time-mated females, where the observation of the copulation plug was taken as E0.5. Collected embryos were immersion-fixed in 4% paraformaldehyde/PBS at 4°C for 3 h. All samples were then cryoprotected in 30% sucrose overnight or until they sank, then frozen in O.C.T (Tissue-TeK) using dry ice, and kept at −80°C. Samples were serially cryosectioned using CM3050S Leica cryostat and collected on Superfrost plus slides (VWR) at 14 μm for immunostainings and 18 μm for *in situ Hybridizations (ISH)*. All slides were stored at −80°C until ready for staining.

### Immunohistochemistry

Primary antibodies and dilutions used in this study were: chicken-α-peripherin (1:1,500, Abcam, Cambridge, MA, USA), rabbit-α-peripherin (1:2,000, Millipore, Darmstadt, Germany), SW rabbit-α-GnRH-1 (1:6,000, Susan Wray, NIH), rabbit α-LHRH (1:4,000, Immunostar, Hudson, WI, USA); chicken-α-GFP (1:1,000, Abcam). Antigen retrieval was performed in a citric acid solution prior to incubation with chicken-α-peripherin, rabbit-α-GnRH-1. For immunoperoxidase staining, slides with collected sections were first rehydrated in PBS for 5 mins then put into a 0.3% H_2_O_2_ solution (methanol/PBS) to block endogenous peroxidases for 30 mins, the sections were further blocked and permeabilized in a prepared solution (10% horse serum, 1% bovine serum albumin, 0.1% sodium azide, 0.5% Triton in PBS) for ~1 h. Sections were then incubated in primary antibody diluted in dilution buffer (0.5% BSA, 0.1% sodium azide in PBS) overnight at 4°C. After incubating in primary antibody, sections were incubated in a biotin conjugated secondary antibody (Vector) diluted in PBS and 0.5% triton (all secondary antibodies were diluted 1:1,000) for 1–3 h, sections were further incubated in a horse-radish peroxidase (HRP) labeled avidin-biotin complex (Vectastain ABC Kit, Vector) that was prepared in PBS. Detection of the horse-radish peroxidase (HRP) was done using diaminobenzidine (DAB), in a glucose solution with the enzyme glucose oxidase, sections were then counterstained with methyl green. For immunofluorescence, sections were processed as described above with the exclusion of peroxidase inhibition. Species-appropriate secondary antibodies were conjugated with Alexa-488, Alexa-594, or Alexa-568 (Molecular Probes and Jackson Laboratories, Inc., Westgrove PA, USA) as specified in the legends. Sections were counterstained with 4′,6′-diamidino-2-phenylindole (1:3,000; Sigma-Aldrich) and coverslips were mounted with Fluoro Gel (Electron Microscopy Services). Sections were washed in 1× PBS before citrate buffer antigen retrieval, and before incubation in any different solutions. Confocal microscopy pictures were taken on a Zeiss LSM 710 microscope. Epifluorescence pictures were taken on a Leica DM4000 B LED fluorescence microscope equipped with a Leica DFC310 FX camera. Images were further analyzed using FIJI/ImageJ software.

### *In situ* Hybridization

Digoxigenin-labeled cRNA probes were prepared by *in vitro* transcription (DIG RNA labeling kit; Roche Diagnostics, Basel, Switzerland) from the following templates: NELL2 (kindly donated by Dr. Tessier-Lavigne’s group; 7). *In situ* hybridization was performed on 18 μm cryosections that were rehydrated in 1× PBS for 5 min, then fixed in 4% PFA in phosphate buffered saline (PBS) for 20 min at 4°C, treated with 10 μg/mL proteinase K (Roche) in 0.1 M phosphate buffer in for 12 min at 37°C, then re-fixed in 4% PFA at 4°C for 20 min. To inactivate the internal alkaline phosphatase, the tissue was treated with 0.2 M HCl for 30 min at 37°C. Nonspecific binding of the probe to slides was reduced by incubating slides in 0.1 M triethanolamine (pH 8.0)/0.25% acetic anhydride solution for 10 min, then washing with 2× Saline-Sodium Citrate (SSC) buffer before incubating in hybridization solution for 2 h at room temperature. Slides were then hybridized with 200 μl of probe (1:100) in hybridization solution at 65°C overnight in a moisture chamber. After hybridization, the slides were washed in 2× SSC, briefly, then in 1× SSC/50% formamide for 40 min at 65°C. RNase A treatment (10 μg/mL) was carried out at 37°C for 30 min. The slides were then washed with 2× SSC then 0.2 × SSC for 15 min each at 65°C. Hybridization was visualized by immunostaining with an alkaline phosphatase conjugated anti-DIG (1:1,000), and NBT/BCIP developer solution (Roche Diagnostics). After color reaction, the slides were put into 10 mM Tris-HCl pH 8.0/1 mM EDTA. Hybridization was visualized by immunostaining with an alkaline phosphatase conjugated anti-DIG (1:1,000), and NBT/BCIP developer solution (Roche Diagnostics). After color reaction, the slides were put into 10 mM Tris-HCl pH 8.0/1 mM EDTA.

### Mapping the Distribution of GnRH-1 Neurons

Whole heads were cryosectioned at 14 μm thickness. Two non-serial series of each animal were then immunostained against GnRH-1 (SW) in diaminobenzidine (DAB) and counterstained with methyl green. All sections were imaged at 10x in brightfield. Sections were aligned in PhotoShop CS6 using the forebrain junction (FBJ), cortex, and cribiform plate as landmarks. Cell bodies were marked and overlaid, representing a cross section of their migratory path. The coordinates of each cell body was plotted in reference to the origin (*x* = 0; *y* = 0), which was set at the GnRH-1ns entry point, using FIJI. The number of GnRH-1ns distributed along the rostro-caudal and dorso-ventral axes were quantified in 400 μm intervals grid for each animal. Differences at each interval between genotypes was assessed by unpaired *t*-Test.

### Statistical Analyses

All statistical analyses were carried out using GraphPad Prism7 Software. Cell counts were done on serial sections immunostained for GnRH-1 and visualized under bright field (immunoperoxidase) or epi-fluorescence illumination (20× ; Leica DM4000 B LED), according to their anatomical location [i.e., (1) nasal region (VNO, axonal tracks surrounding the olfactory pits); (2) FBJ; and (3) brain (all the cells that accessed the OB and were distributed within the forebrain)]. For each animal, counts were performed on two non-serial series. The average number of cells from these two series was then multiplied by the total number of series/animal to compute a value for each animal, four series at E14.5, five series at E15.5-E17.5. These were then averaged ± standard error (SE) among animals of the same age and genotype. Means ± SEs were calculated on at least three animals per genotype. Cell counts based on distance were performed on 5× images of the same sections in FIJI. The brain was divided into 200 μm sections where the originating distance is the point of entry for the GnRH-1ns (ventral to the OBs) into the forebrain and the farthest distance being the median eminence located in the most ventral portion of the brain. The statistical difference between genotypes and groups were determined using *t*-test, *p* values were corrected using the Holm Šídák method. All data are represented as the mean ± SEM from *n* ≥ 3 mice per genotype/age for each experiment. Values of *p* < 0.05 were considered statistically significant.

## Results

### NELL2 Is Expressed in the Developing Brain While Robo3 Is Expressed by GnRH-1 and TN Fibers

**Figure 1:**
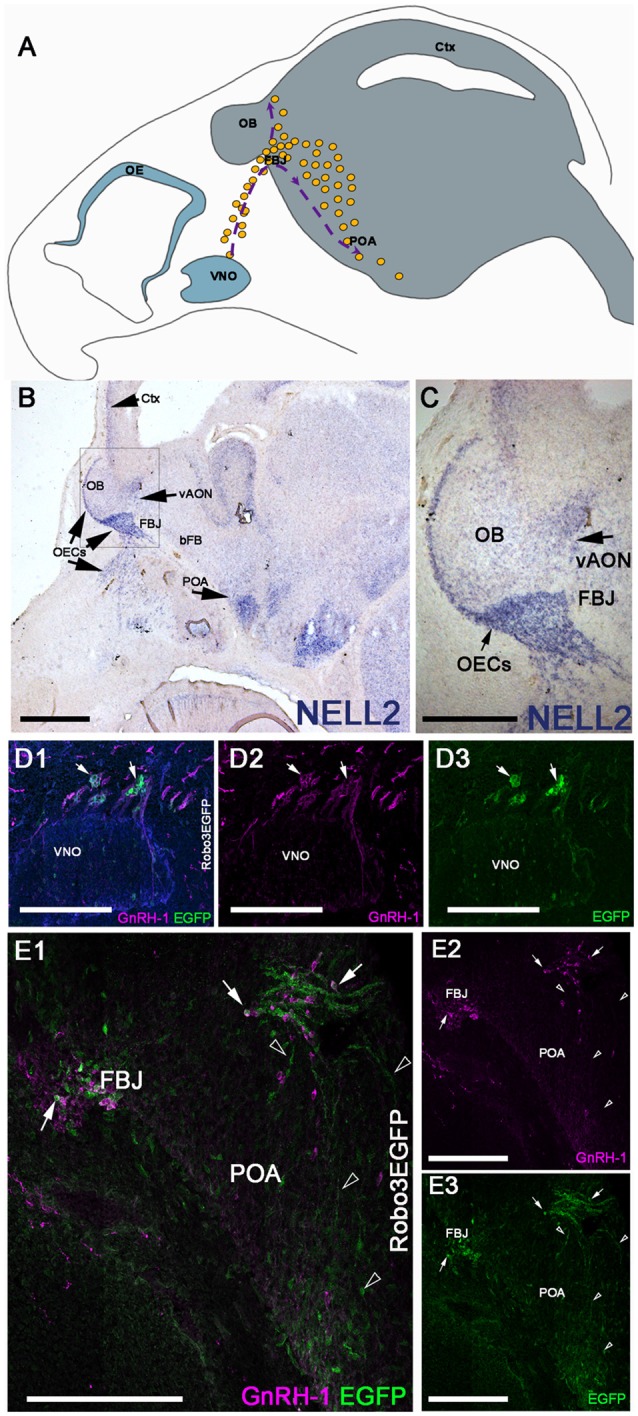
Neural EGFL Like 2 (NELL2) is expressed along the gonadotropin releasing hormone-1 (GnRH-1) migratory path, while roundabout 3 (Robo3) is expressed by the terminal nerve (TN) and some GnRH-1 neurons. **(A)** Schematic representing the head of a E15 mouse embryo. The olfactory epithelium (OE) and vomeronasal organ (VNO) are represented in blue in the nasal area. The GnRH-1 neurons (yellow dots) originating from the VNO are shown on their migratory route, indicated by purple dotted lines and arrows. The GnRH-1ns enter the brain (gray) at the level of the forebrain junction (FBJ) where a small number of cells migrate around the olfactory bulb (OB) while the majority of cells migrate to the pre-optic area of the hypothalamus (POA; **B**). *In situ* hybridization against NELL2 on E14.5 shows NELL2 expression in the cortex (Ctx), OBs, ventral anterior olfactory nucleus (vAON), olfactory ensheathing cells (OECs) and the POA. **(C)** Magnification of boxed area in **(A)**.**(D)** E14.5 immunostaining against GnRH-1 and GFP on Robo3EGFP^+/−^ knock in mouse embryo. GnRH-1 neurons (magenta) and Robo3EGFP+ positive cells (putative TN cells) migrate out of the VNO. Nuclei are stained in blue. **(E1–3)** GnRH-1 neurons access the brain from the FBJ along Robo3EGFP positive fibers of the putative TN (arrowheads). Robo3EGFP expression in some GnRH-1 cells (arrows), see single channels in **(D2,3)**. Scale bars are 500 μm in **(B)**, 250 μm in **(C)**, and 200 μm in **(D1–E3)**.

NELL2 is a diffusible ligand that binds and repels Robo3 expressing neurons (Jaworski et al., 2015). When GnRH-1ns migrate from the nasal area to the hypothalamus at E14.5, we found NELL2 expression in the cortex and along the GnRH-1ns migratory track, in the olfactory ensheathing cells (OECs), OBs, ventral anterior olfactory nucleus (vAON), the FBJ, where the GnRH-1 neurons invade the brain, and in the preoptic area (POA; Figures 1A,B). Robo3 is a multifunctional molecule that integrates attractive and repulsive signals of Netrin, Slits, and NELL2 (Marillat et al., 2004; Sabatier et al., 2004; Camurri et al., 2005; Chen et al., 2008; Zelina et al., 2014; Jaworski et al., 2015; Friocourt and Chédotal, 2017), and has been previously reported in migratory neurons proximal to the vomeronasal organ (putative TN cells) and in some migratory GnRH-1ns (Cariboni et al., 2012). Taken together, we hypothesized that NELL2 may contribute to the overall migration of GnRH-1ns toward the basal forebrain (bFB). By using Robo3GFP knock-in mice we confirmed detectable Robo3 expression in some of the GnRH-1 immunoreactive cells emerging from the vomeronasal organ, and by other GnRH-1 negative cells of the migratory mass (Figures 1D1–3). Moreover, we were able to visualize GnRH-1 neurons invading the brain along Robo3 expressing fibers of the putative TN (Taroc et al., 2017; Figures 1E1–3).

### GnRH-1 Neuronal Migration Is Not Affected by NELL2 Loss-of-Function

**Figure 2:**
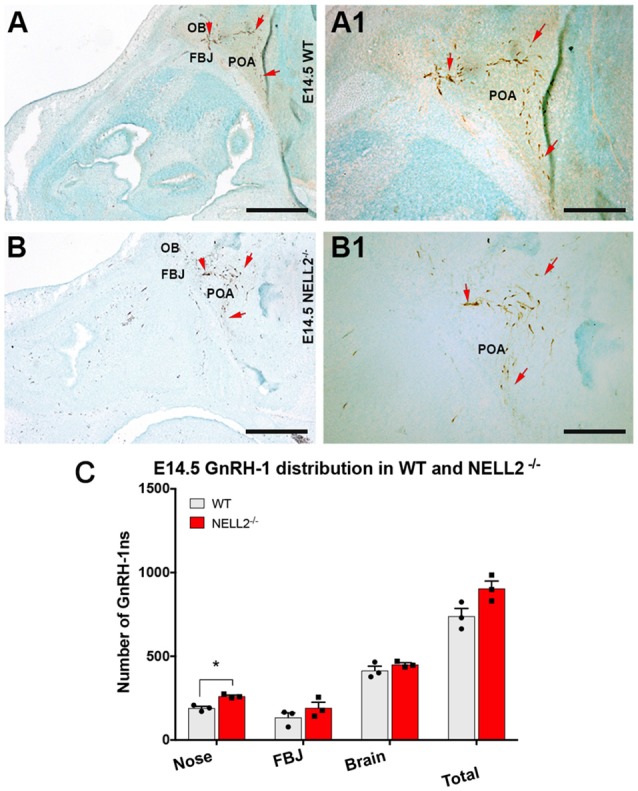
Lack of functional NELL2 does not compromise GnRH-1 neuronal migration to the preoptic area (POA). E14.5, GnRH-1 immunostaining, representative images of GnRH-1 neurons migrating in **(A,A1)** controls and **(B,B1)** NELL2^−/−^ animals. **(A1,B1)** In both control and mutant animals, GnRH-1 neurons, as indicated by the red arrows, make a sharp turn towards the basal forebrain (bFB). **(C)** Quantification of GnRH-1 neurons distribution in nasal area, FBJ, brain, and whole head. Significantly higher number of GnRH-1 immunoreactive cells was found in the nasal area of NELL2 mutants (*p = 0.04), there is also a non-significant higher number of GnRH-1 positive cells in the FBJ but no differences were found in the brain. Means ± SEM, each dot represents one animal. Scale bars are 500 μm in **(A,B)**, and 250 μm in **(A1,B1)**.

Based on the expression of Robo3 and NELL2 along the GnRH-1ns migratory route, the reported phenotype of Robo3^null^ mutants (Cariboni et al., 2012) and the ligand-receptor relationship of NELL2 and Robo3 (Jaworski et al., 2015), we analyzed GnRH-1 neuronal migration in NELL2^−/−^ animals. In NELL2 mutants, GnRH-1ns successfully invaded the brain ventral to the OBs and migrated towards the bFB. Analysis at E14.5 revealed a small increase in the number of GnRH-1 immunoreactive cells in the nasal area of NELL2^−/−^ mutants. However, we found no significant differences in the number of cells in the brain between controls and NELL2 mutants (Figure 2C). This analysis suggests lack of a central role for NELL2 in controlling GnRH-1ns ability to access to the bFB.

### GnRH-1 Neuronal Migration Remains Unperturbed in Robo3^−/−^ Mutants

**Figure 3:**
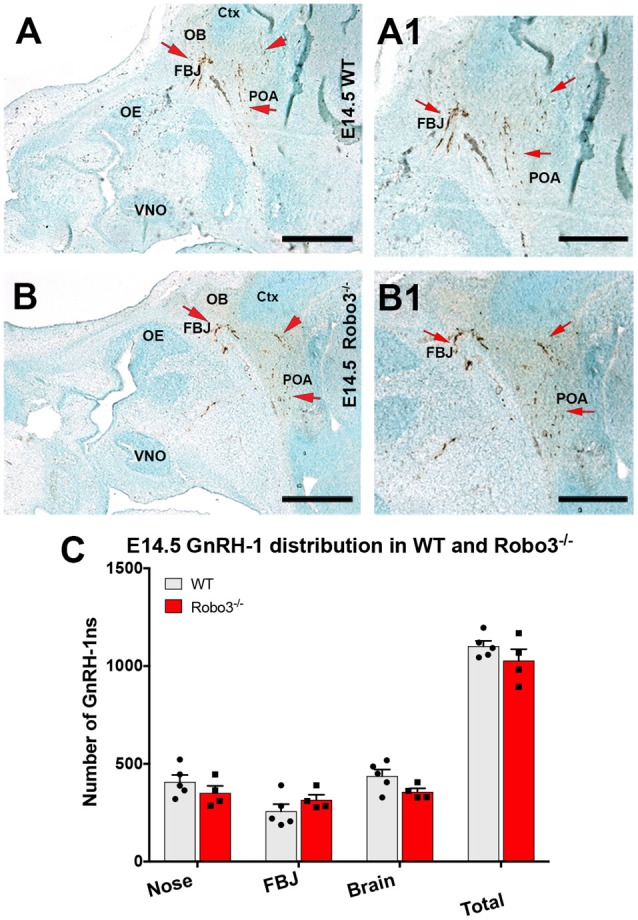
Lack of functional Robo3 does not compromise GnRH-1 neuronal migration. E14.5, Anti GnRH-1 immunostaining, GnRH-1 neurons migrating in controls (**A,A1)** and Robo3 KO **(B,B1)**. **(A1,B1)** In both control and mutant animals, GnRH-1 neurons turn towards the bFB. **(C)** Quantification of GnRH-1 neurons distribution in nasal area, FBJ, brain and whole head. Mean ± SEM, each dot represents one animal. Scale bars are 500 μm in **(A,B)**, and 250 μm in **(A1,B1)**.

As we observed no major abnormalities for the GnRH-1ns in NELL2 mutants, we decided to compare GnRH-1 neuronal migration of NELL2^−/−^ with Robo3^−/−^ mutants (Jaworski et al., 2015). Robo3^−/−^ mutants and wild type (WT) controls were analyzed at E14.5, the same stage previously used for NELL2^−/−^ mutants. Surprisingly, and in contrast to previously reported findings (Cariboni et al., 2012), our quantifications indicated no defective GnRH-1 neuronal migration in Robo3^−/−^ mutants, as no differences were found in total cell number nor in cell distribution between the nasal area and brain (Figure 3).

### GnRH-1 Neuronal Migration Is Not Affected in NELL2^−/−^/Robo3^+/−^ Double Mutants

**Figure 4:**
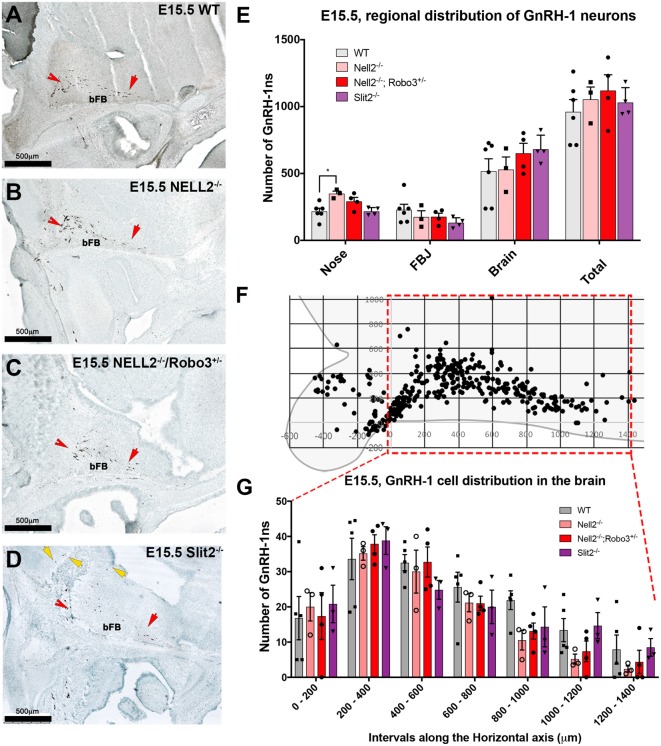
Comparable GnRH-1 neuronal migration between controls and NELL2^−/−^, NELL2^−/−^/Robo3^+/−^, and Slit2^−/−^ mutants. E15.5, Anti GnRH-1 immunostaining, GnRH-1 neurons migrating in E15.5 wild type (WT) controls **(A)**, NELL2^−/−^ **(B)**, NELL2^−/−^/Robo3^+/−^ **(C)** and Slit2^−/−^ mutants **(D)**. In control and all the mutant animals, GnRH-1 (Red notched arrows) neurons migrated to the bFB towards the POA (red arrow). In Slit2 null mutants, more GnRH-1 cells migrated dorsally (yellow arrows). **(E)** Quantification of GnRH-1 neurons distribution in nasal area, FBJ, brain and total number of GnRH-1 immunoreactive cells in the whole head. **(F)** Cartoon representing GnRH-1 cell distribution in a WT brain in different dorso-ventral and rostro-caudal intervals, 200 μm interval grid. (**G**) Distribution of the GnRH-1 neurons in the brain in the indicated genotypes. Each bar indicates the average of total number of cells within 200 μm interval along the X-axis use boxed area in **(F)** as a reference. Means ± SEM (*p ≤ 0.05). Distance was measured setting the entry point of the GnRH-1 neurons in the brain. Scale bars are 500 μm in all images.

To understand how NELL2 and Robo3 contribute to controlling GnRH-1 neuronal migration, we generated and analyzed NELL2^−/−^/Robo3^+/−^ double mutants against NELL2^−/−^ single mutants and WT controls at E15.5 (Jaworski et al., 2015). As in E14.5 mutants, the E15.5 NELL2^−/−^ single mutants, contrary to what is suggested by the literature (Ha et al., 2008; Zhou and Li, 2014), had a small but significant increase in the number of GnRH-1 immunoreactive cells in the nasal region when compared with WT controls. However, the NELL2^−/−^/Robo3^+/−^ double mutants did not show any significant differences in cell distribution between the nasal area and brain compared to controls, as GnRH-1ns could migrate towards the hypothalamic area in a comparable fashion as controls (Figures 4A–C,E). These data suggest NELL2-Robo3 ligand-receptor axis does not control GnRH-1ns motility and migration to the bFB.

### Some GnRH-1 Neurons Show Aberrant Routing in Slit2^−/−^ Mutants

Since we did not observe significant defects in GnRH-1ns migration to the brain in Robo3^−/−^, NELL2^−/−^, or NELL2^−/−^/Robo3^+/−^ mutants, we analyzed Slit2^−/−^ mutants to test if the reported GnRH-1ns migratory defects in Slit2 null mice result from an alternative mechanism from the proposed Slit2-Robo3 interaction (Cariboni et al., 2012). Counting the GnRH-1ns distributed between the nasal area and the brain at E15.5 did not show a significant difference in the number of GnRH-1ns in the brain of Slit2 null mutants compared to WT controls, nor an accumulation of GnRH-1ns in the nasal area (Figure 4E). We then analyzed the distribution of GnRH-1ns in the brain from the entry point ventral to the OBs to the POA. This analysis revealed comparable GnRH-1 cell migration in E15.5 WT, Robo3^−/−^, NELL2^−/−^, NELL2^−/−^/Robo3^+/−^, and Slit2^−/−^ animals (Figure 4G).

**Figure 5:**
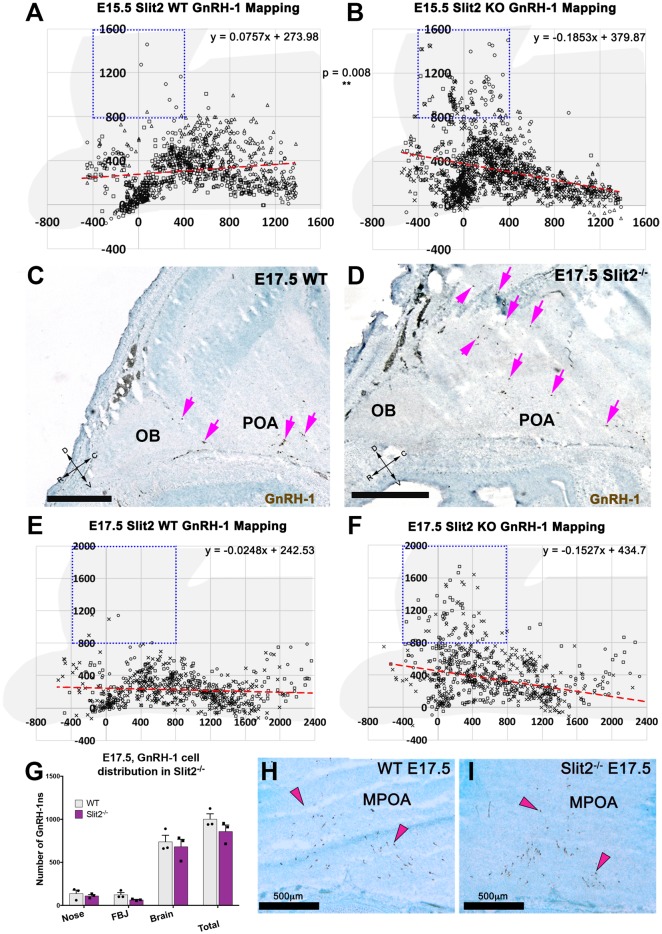
Slit2 mutants display a different GnRH-1 cell patterning in the brain. **(A,B)** Plots representing the distribution of GnRH-1 neurons in the brain, at E15.5. Cell position was recorded for 355 ± 8 cells/animal of in three WT **(A)** and three Slit2 KO** (B)** animals. Use brain silhouette in light gray for spatial reference. Each square represents a 400 × 400 μm interval. The boxed area indicates the regions where significant difference was found between genotypes. Red dashed lines representing linear regressions of the scatterplots, equations on top right, indicate different cell in the brain patterning between genotypes. Different marker shapes indicate cells plotted from different animals. **(C,D)** E17.5 representative images showing larger number of GnRH-1 neurons (magenta arrows) distributed dorsally in Slit2 null mutants **(D)** compared to control **(C,E,F)**. E17.5, plots representing the distribution of GnRH-1 neurons in the brain, cell position was recorded for 194 ± 10 cells/animal of three WT **(E)** and three Slit2 KO. Use brain silhouette in light gray for spatial reference; different markers indicate cells plotted from different animals. Boxed area indicates area where significant difference was found in GnRH-1 cell number between genotypes. Trend lines equations on top right, indicate different cellular patterning between genotypes as observed at E15.5, compare to **(H,I)**. **(G)** Distribution of GnRH-1 neurons between nasal area and brain of controls and Slit2 mutants and at E17.5. **(H,I)** Representative images showing comparable immunoreactivity and number of GnRH-1 neurons in the medial POA (MPOA) of WT **(H)** and Slit2 KOs at E17.5 **(I)**. Scale bars are 500 μm in all images.

Upon visual inspection during quantification, we noticed that the GnRH-1ns in the Slit2^−/−^ animals appeared to follow a different migratory pattern in the brain from controls (Figure 4D). We plotted the position of GnRH-1ns in WTs (*n* = 3) and Slit2 mutants (*n* = 4; Figure 5). At E15.5, we observed a significant increase in the number of GnRH-1ns migrating dorsally towards the cortical region of the brain (*n* = 4; *p* = 0.006; See boxed area in Figures 5A,B). Despite this apparent misrouting of a subset of GnRH-1ns into the cortical region, we observed non-significant decrease in the number of GnRH-1ns in the bFB (WT 737.5 ± 12.99; Slit2^−/−^ 600.8 ± 57.01; *p* = 0.08). Using linear regression of the plots representing the position of GnRH-1ns in the brain, we identified comparable cell numbers along the migratory route, although GnRH-1ns in Slit2 mice followed a different trajectory from control animals (Figures 5A,B).

As we observed differences in the GnRH-1ns routing in Slit2 null animals at E15.5, we sought to further analyze these animals at E17.5 (Figures 5C–I), the developmental stage at which GnRH-1 neuronal migration is virtually completed. We confirmed cell counts in GnRH-1ns in the brain were comparable between Slit2 mutants and controls (Figure 5G). However, a significant portion of the GnRH-1ns appeared ectopically positioned in the cortical area as seen at E15.5 (Figures 5C–F). Linear regression of the scatter plots at E15.5 and E17.5 suggest similar differences in patterning between controls and mutants at both analyzed stages. GnRH-1ns distribution in the bFB did not appear significantly altered in Slit2 mutants (WT: 589 ± 50.92; Slit2^−/−^ 574 ± 127.4; *p* = 0.9). These data suggest that without Slit2, GnRH-1ns successfully migrate into the brain. However, once in the brain lack of Slit2 alters the migratory trajectory of the GnRH-1ns (Figure 5).

### GnRH-1 Cell Distribution Remains Constant Using Antibodies Against the Mature Form of GnRH/LHRH

**Figure 6:**
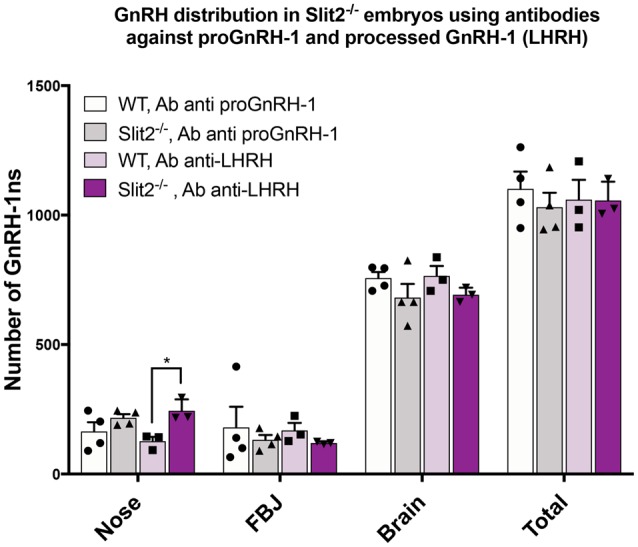
Analysis of Slit2 mutants with two anti GnRH-1 antibody corroborates comparable number of GnRH-1 in the brain compared to controls. Quantification of GnRH-1 immunoreactive cells on serial sections of WT and Slit2^−/−^ mutants at E17.5 in the nose, FBJ and brain. Two alternate serial sections of the same animals were stained with either anti proGnRH-1 (SW) or anti mature GnRH-1 (LHRH) antibodies. Each dot indicates independent animal, values ± SEM. No statistical differences in the number of neurons per area were found based on the antibody used, however, the anti-LHRH antibody yielded a significant increase of immunoreactive cells in the nasal region that was not observed in the anti-GnRH (*p ≤ 0.05).

The previously published analyses of GnRH-1 neuronal motility in Robo3 and Slit2 null mutants (Cariboni et al., 2012) used an antibody against the mature, cleaved and amidated form of GnRH-1, also known as LHRH (luteinizing hormone-releasing hormone; Baba et al., 1971; Matsuo et al., 1971; Cariboni et al., 2005, 2012). We performed all the previously described experiments using the SW antibody that recognizes the immature, unprocessed form of GnRH-1, proGnRH-1 (Wray et al., 1989a). During embryonic development, the mature form of GnRH-1/LHRH may be differentially expressed within the GnRH-1 neuronal population (Livne et al., 1993). So, we repeated the experiments in the Slit2 mutant line using the anti-LHRH antibody to understand if the lack of Slit2 altered proGnRH-1 processing rather than GnRH-1 cell migration (Livne et al., 1993). We performed immunohistochemistry using anti-LHRH on serial sections from the same animals previously labeled with SW at E15.5 (Cariboni et al., 2012). We found that the anti-LHRH antibody labeled an almost identical number and distribution of GnRH-1ns in both Slit2 null and WT control mice (Figure 6). Interestingly, we observed a significantly increased number of cells immunoreactive for LHRH in the nasal area of Slit2 null animals when compared to the WT control stained with LHRH. However comparable numbers of GnRH-1+ and LHRH+ cells were found in all other regions (Figure 6). Based on this data, we conclude that the differences between our analysis and published work (Cariboni et al., 2012) may derive from other technical issues.

## Discussion

HH and KS are developmental disorders that negatively impact sexual maturation and fertility. Though several single-gene mutations have been linked to HH and KS a growing number of evidence suggests a complex multigenic nature for these pathologies (Pitteloud et al., 2007, 2010).

A previous study suggested that single mutations in Robo3 or Slit2 could severely impair GnRH-1 neuronal motility and migration to the brain (Cariboni et al., 2012). However, our analysis on multiple mouse models carrying NELL2, Robo3, NELL2/Robo3, and Slit2 loss-of-function did not reveal any significant defects of GnRH-1 neuronal motility/migration. Moreover, we did not identify a crucial role for NELL2 in controlling GnRH-1ns trajectory to the brain. Our results challenge proposals that monogenic mutations in Robo3 or Slit2 are sufficient to affect GnRH-1 cell motility.

During embryonic development, GnRH-1ns follow a long and well-defined route from the olfactory placode to the bFB. After being born in the nasal pit between E10.5 and E11.5, GnRH-1ns migrate along TN fibers across the nasal mesenchyme into the forebrain (Wray et al., 1989a,b; Forni et al., 2011; Forni and Wray, 2015). After crossing the cribriform plate, GnRH-1ns steer ventrally towards the hypothalamus while extending long processes toward the median eminence (Reed et al., 2002; Schwarting et al., 2004, 2006, 2007). In the last 20 years, several groups have focused on identifying cues responsible for GnRH-1 neuronal migration and routing (Wierman et al., 2011; Forni and Wray, 2015), as defects in GnRH-1ns migration can cause impaired fertility and pubertal onset.

The Slit proteins, Slit1 and 2, and their guidance receptors, Robo1 and Robo2, play a pivotal role in routing axons of commissural neurons in the spinal cord and controlling olfactory system development and axonal fasciculation (Nguyen Ba-Charvet et al., 1999, 2001, 2002; Marillat et al., 2002, 2004; Cloutier et al., 2004; Sabatier et al., 2004; Jaworski and Tessier-Lavigne, 2012). The receptor Robo3 plays a complex role in axonal guidance by silencing Robo1 and 2-mediated Slit repulsion, which increases DCC-mediated sensitivity to Netrin attraction and triggers axonal repulsion in response to its ligand NELL2 (Marillat et al., 2004; Chen et al., 2008; Zelina et al., 2014; Jaworski et al., 2015).

Dramatic migratory defects of GnRH-1ns have been described in Robo3 and Slit2 null mutant mice on C57 background suggesting that a Robo3-Slit2 guidance axis can control GnRH-1 cell motility (Cariboni et al., 2012). However, no further evidence supported a direct binding of Slit2 to Robo3 (Camurri et al., 2005; Zelina et al., 2014; Jaworski et al., 2015), while NELL2 has been identified as a direct ligand for Robo3 (Jaworski et al., 2015). Surprisingly, our results on mutant mouse lines did not confirm effects of monogenic deletions in Robo3 or Slit2 in controlling GnRH-1ns ability to migrate into the brain. In addition, with the exception of a small increase of GnRH-1 immunoreactivity in the nasal area (Figures 2, 4), we did not find significant defects after NELL2 loss-of-function. Notably, the small difference observed in the NELL2^−/−^ mutants in the nasal area was not detected in the NELL2^−/−^/Robo3^+/−^ double mutants, making us wonder if such difference may represent small developmental variances between littermates rather than genotypic differences.

Some GnRH-1ns and TN fibers express Robo3 (Figure 1). Sources of Slit proteins are believed to prevent olfactory fibers from invading the brain (Nguyen-Ba-Charvet et al., 2008; Taroc et al., 2017). However, our data suggest that Robo3 is not required to silence potential Slit protein repulsive signaling in GnRH-1ns or TN fibers. In addition, NELL2-Robo3 mediated axonal repulsion at the FBJ and proximal to the OBs (Figure 1) is not required to control GnRH-1ns migratory trajectory to the bFB (Figure 4). Since our analysis of Slit2 null mutants did not find migratory defects of GnRH-1ns to the brain (Figure 4), our results challenge a previously proposed role for Slit2 to control GnRH-1 cell motility, alone or through Robo3 (Cariboni et al., 2012). However, the GnRH-1ns migratory route appears to change in Slit2 null mutants despite no obvious migratory defects to reach the brain (Figure 5). Rather, we observed a different GnRH-1 neuronal patterning in the brain of Slit2 null mutants with increased GnRH-1ns migrating dorsally towards the cortex. This is an intriguing phenotype, since a previous study failed to detect Robo1 and Robo2 expression in the TN and GnRH-1ns (Taroc et al., 2017). Consistent with this, no GnRH-1 neuronal migratory or patterning defects were reported in Robo1/Robo2 double mutants (Cariboni et al., 2012).

Robo1 and Robo2 are the primary receptors mediating Slit activities. Surprisingly, Robo1/Robo2 double mutants have no GnRH-1 neuronal migratory defects (Cariboni et al., 2012) and we did not find phenotypical overlaps between Slit2 and Robo3 mutants. Based on these data, we are inclined to exclude a key role for Robo receptors in controlling GnRH-1ns migration. However, deficiencies in Robo1, Robo2, and all Slits (Slit1, Slit2 and Slit3) do not give completely overlapping phenotypes in spinal commissural axonal guidance (Jaworski et al., 2010; Delloye-Bourgeois et al., 2015). These results suggested that additional receptors, such as PlexinA1 (Delloye-Bourgeois et al., 2015), may contribute to Slit protein mediated guidance.

The reasons for such dramatic discrepancies among our phenotypes and the previously published analyses of Robo3 and Slit2 mutants (Cariboni et al., 2012) remain unknown. Notably, a recent study also reported normal GnRH-1 neuronal migration in *Reelin*, Reeler mutant mice, (Dairaghi et al., 2018) contradicting the findings of dramatic migratory defects for GnRH-1ns in the same mouse line (Cariboni et al., 2005). Disagreements among studies can arise from technical issues like different sensitivities of antibodies. However, we analyzed GnRH-1 neuronal migration in Slit2 null mutants with two distinct antibodies: one that recognizes the unprocessed form of GnRH-1 (SW; Wray et al., 1989b) and another that binds to the mature form of GnRH-1 (LHRH; Cariboni et al., 2005). This approach yielded similar results, indicating comparable GnRH-1 neuron migratory rates from the nose to the brain between Slit2 KO and controls.

A variable that we cannot exclude is the genetic background of the analyzed animals. All the experiments here were conducted on mice on CD-1 background, which is an outbred strain, while the study by Cariboni et al. (2012) analyzed Robo3 and Slit2 mutants on C57BL/6 inbred mouse strain. There are multiple published examples of different phenotypic outcomes arising from the same genetic mutations using mice from different backgrounds (Dixon and Dixon, 2004; Errijgers et al., 2008; Sisay et al., 2013; Coley et al., 2016; Mahajan et al., 2016).

Analysis of the CD-1 mouse genome reported patterns of linkage disequilibrium and heterogeneity close to the one of mice caught in the wild (Aldinger et al., 2009; Zhang et al., 2012). However, our unpublished observations demonstrate that Robo3 and Slit2 mutants are perinatal lethal on CD-1 background as they are on C57B. Moreover, CD-1 mice are sensitive to Slit1/Slit2 and to NELL2^−/−^/Robo3^+/−^ axonal misrouting, in the neural tube, as reported for other genetic backgrounds (Jaworski et al., 2015). The fact that in CD-1 mice some of the known abnormal phenotypes of Slit2 and Robo3 KO are found, but not GnRH-1 migratory defects, could suggest that the regulation of GnRH-1 migration and routing is more robust than that of other processes. If Robo3 and Slit2 loss-of-function cause migratory impairments for GnRH-1ns on a C57BL/6 background but not on CD-1, this would imply that the inbred C57B is a sensitized strain that must carry variants in other genes, involved in GnRH-1 neuronal migration. If phenotypes on C57BL/6 will be further confirmed this would make this specific mouse strain an interesting model to dissect the role of genetic variants and modifiers in the oligogenic nature of KS and HH (Montagutelli, 2000).

A small number of GnRH-1 cells, between 12 and 34%, are required to trigger puberty and for cyclical control in adult female mice (Herbison et al., 2008). Our data on CD-1 mice suggest that none of the analyzed mutations along the Robo1/2-Slit2 and Robo3-NELL2 guidance axis are sufficient, in an outbred mouse background, to cause GnRH-1 migratory deficiencies that would translate into HH.

Netrin can control GnRH-1ns routing ventrally to the median eminence (Schwarting et al., 2004). Robo3 can increase DCC-mediated sensitivity for Netrin attraction (Zelina et al., 2014). However, we did not observe similar phenotypes in our Robo3 KO to those described in the Netrin KO. This suggests that Robo3 contributions to Netrin signaling is dispensable for the development of the GnRH-1 neuronal system. Netrin may also induce GnRH-1 neurite outgrowth in the median eminence (Low et al., 2012). Further detailed analyses will establish if Robo3 contributes to the Netrin mediated axonal growth of GnRH-1ns. Since we found that migration of GnRH-1ns to the brain is not impaired in Robo3^−/−^, NELL2^−/−^, NELL2^−/−^/Robo3^+/−^, and Slit2^−/−^ mutants, we conclude that the GnRH-1 cell motility or access to the brain does not depend on these genes. However, we did find that Slit2 loss-of-function altered normal GnRH-1 cell distribution in the brain. Future, studies will elucidate the molecular mechanisms underlying the trajectory defects and cell autonomy of patterning defects in Slit2 mutants. In conclusion, we analyzed the role of the NELL2-Robo3 signaling axis in controlling GnRH-1 neuronal migration from the nose to the hypothalamic area. Though Robo3 is expressed by some GnRH-1ns and cells of the putative TN (Figure 1; Taroc et al., 2017) and NELL2 was expressed along the GnRH-1ns migratory path, we found no significant effects after loss-of-function of these genes on GnRH-1 neuronal migration on CD-1 mice. What role the multifunctional receptor Robo3 plays in the GnRH-1 system, remains an important and unresolved question.

## Author Contributions

PF conceptualized and designed the experiments and analyzed the data. ET, JL and AT performed the experiments and analyzed the data. AJ provided reagents and designed the experiments. ET, JL, and PF wrote the manuscript.

## Conflict of Interest Statement

The authors declare that the research was conducted in the absence of any commercial or financial relationships that could be construed as a potential conflict of interest.

## References

[B1] Aldinger K. A. Sokoloff G. Rosenberg D. M. Palmer A. A. Millen K. J. (2009). Genetic variation and population substructure in outbred CD-1 mice: implications for genome-wide association studies. PLoS One 4:e4729. 10.1371/journal.pone.0004729 19266100 PMC2649211

[B2] Andrews W. D. Barber M. Parnavelas G. J. (2007). Slit-Robo interactions during cortical development. J. Anat. 211, 188–198. 10.1111/j.1469-7580.2007.00750.x 17553100 PMC2375773

[B3] Baba Y. Matsuo H. Schally V. A. (1971). Structure of the porcine LH- and FSH-releasing hormone. II. Confirmation of the proposed structure by conventional sequential analyses. Biochem. Biophys. Res. Commun. 44, 459–463. 10.1016/0006-291x(71)90623-1 4946067

[B4] Balasubramanian R. Choi J. H. Francescatto L. Willer J. Horton E. R. Asimacopoulos E. P.. (2014). Functionally compromised CHD7 alleles in patients with isolated GnRH deficiency. Proc. Natl. Acad. Sci. U S A 111, 17953–17958. 10.1073/pnas.1417438111 25472840 PMC4273325

[B5] Barraud P. St John J. A. Stolt C. C. Wegner M. Baker V. C. (2013). Olfactory ensheathing glia are required for embryonic olfactory axon targeting and the migration of gonadotropin-releasing hormone neurons. Biol. Open 2, 750–759. 10.1242/bio.20135249 23862023 PMC3711043

[B6] Boehm U. Bouloux P. M. Dattani M. T. de Roux N. Dodé C. Dunkel L.. (2015). Expert consensus document: european consensus statement on congenital hypogonadotropic hypogonadism—pathogenesis, diagnosis and treatment. Nat. Rev. Endocrinol. 11, 547–564. 10.1038/nrendo.2015.112 26194704

[B7] Camurri L. Mambetisaeva E. Davies D. Parnavelas J. Sundaresan V. Andrews W. (2005). Evidence for the existence of two Robo3 isoforms with divergent biochemical properties. Mol. Cell. Neurosci. 30, 485–493. 10.1016/j.mcn.2005.07.014 16226035

[B8] Cariboni A. Andrews W. D. Memi F. Ypsilanti A. R. Zelina P. Chedotal A.. (2012). Slit2 and Robo3 modulate the migration of GnRH-secreting neurons. Development 139, 3326–3331. 10.1242/dev.079418 22912413 PMC3424043

[B9] Cariboni A. Davidson K. Dozio E. Memi F. Schwarz Q. Stossi F.. (2011). VEGF signalling controls GnRH neuron survival via NRP1 independently of KDR and blood vessels. Development 138, 3723–3733. 10.1242/dev.063362 21828096 PMC3152927

[B10] Cariboni A. Rakic S. Liapi A. Maggi R. Goffinet A. Parnavelas G. J. (2005). Reelin provides an inhibitory signal in the migration of gonadotropin-releasing hormone neurons. Development 132, 4709–4718. 10.1242/dev.02033 16207762

[B11] Cariboni A. Valentina A. Davidson K. Parnavelas J. (2015). The molecular control of GnRH neuron development. Springerplus 4:L46. 10.1186/2193-1801-4-S1-L46 27386208 PMC4798131

[B12] Casoni F. Hutchins B. I. Donohue D. Fornaro M. Condie B. G. Wray S. (2012). SDF and GABA interact to regulate axophilic migration of GnRH neurons. J. Cell Sci. 125, 5015–5025. 10.1242/jcs.101675 22976302 PMC3533389

[B13] Chen Z. Gore B. B. Long H. Ma L. Tessier-Lavigne M. (2008). Alternative splicing of the Robo3 axon guidance receptor governs the midline switch from attraction to repulsion. Neuron 58, 325–332. 10.1016/j.neuron.2008.02.016 18466743

[B14] Cloutier J. F. Sahay A. Chang E. C. Tessier-Lavigne M. Dulac C. Kolodkin A. L.. (2004). Differential requirements for semaphorin 3F and Slit-1 in axonal targeting, fasciculation and segregation of olfactory sensory neuron projections. J. Neurosci. 24, 9087–9096. 10.1523/JNEUROSCI.2786-04.2004 15483127 PMC6730055

[B15] Coley W. D. Bogdanik L. Vila M. C. Yu Q. Van Der Meulen J. H. Rayavarapu S.. (2016). Effect of genetic background on the dystrophic phenotype in mdx mice. Hum. Mol. Genet. 25, 130–145. 10.1093/hmg/ddv460 26566673 PMC4690497

[B16] Dairaghi L. Flannery E. Giacobini P. Saglam A. Saadi H. Constantin S.. (2018). Reelin can modulate migration of olfactory ensheathing cells and gonadotropin releasing hormone neurons via the canonical pathway. Front. Cell. Neurosci. 12:228. 10.3389/fncel.2018.00228 30127721 PMC6088185

[B17] Delloye-Bourgeois C. Jacquier A. Charoy C. Reynaud F. Nawabi H. Thoinet K.. (2015). PlexinA1 is a new Slit receptor and mediates axon guidance function of Slit C-terminal fragments. Nat. Neurosci. 18, 36–45. 10.1038/nn.3893 25485759

[B18] Dixon J. Dixon J. M. (2004). Genetic background has a major effect on the penetrance and severity of craniofacial defects in mice heterozygous for the gene encoding the nucleolar protein Treacle. Dev. Dyn. 229, 907–914. 10.1002/dvdy.20004 15042714

[B19] Dodé C. Hardelin P. J. (2009). Kallmann syndrome. Eur. J. Hum. Genet. 17, 139–146. 10.1038/ejhg.2008.206 18985070 PMC2986064

[B20] Errijgers V. Fransen E. D’Hooge R. De Deyn P. P. Kooy F. R. (2008). Effect of genetic background on acoustic startle response in fragile X knockout mice. Genet. Res. 90, 341–345. 10.1017/s0016672308009415 18840308

[B22] Forni P. E. Bharti K. Flannery E. M. Shimogori T. Wray S. (2013). The indirect role of fibroblast growth factor-8 in defining neurogenic niches of the olfactory/GnRH systems. J. Neurosci. 33, 19620–19634. 10.1523/JNEUROSCI.3238-13.2013 24336726 PMC3858631

[B23] Forni P. E. Fornaro M. Guenette S. Wray S. (2011). A role for FE65 in controlling GnRH-1 neurogenesis. J. Neurosci. 31, 480–491. 10.1523/JNEUROSCI.4698-10.2011 21228158 PMC3586531

[B21] Forni P. E. Wray S. (2015). GnRH, anosmia and hypogonadotropic hypogonadism—where are we? Front. Neuroendocrinol. 36C, 165–177. 10.1016/j.yfrne.2014.09.004 25306902 PMC4703044

[B24] Friocourt F. Chédotal A. (2017). The Robo3 receptor, a key player in the development, evolution, and function of commissural systems. Dev. Neurobiol. 77, 876–890. 10.1002/dneu.22478 28033646

[B25] Ha C. M. Choi J. Choi E. J. Costa M. E. Lee B. J. Ojeda R. S. (2008). NELL2, a neuron-specific EGF-like protein, is selectively expressed in glutamatergic neurons and contributes to the glutamatergic control of GnRH neurons at puberty. Neuroendocrinology 88, 199–211. 10.1159/000139579 18547942

[B26] Hardelin J. P. Dodé C. (2008). The complex genetics of Kallmann syndrome: KAL1, FGFR1, FGF8, PROKR2, PROK2, et al. Sex Dev. 2, 181–193. 10.1159/000152034 18987492

[B27] Herbison A. E. Porteous R. Pape J. R. Mora J. M. Hurst R. P. (2008). Gonadotropin-releasing hormone neuron requirements for puberty, ovulation, and fertility. Endocrinology 149, 597–604. 10.1210/en.2007-1139 18006629 PMC6101186

[B29] Jaworski A. Long H. Tessier-Lavigne M. (2010). Collaborative and specialized functions of Robo1 and Robo2 in spinal commissural axon guidance. J. Neurosci. 30, 9445–9453. 10.1523/JNEUROSCI.6290-09.2010 20631173 PMC6632452

[B28] Jaworski A. Tessier-Lavigne M. (2012). Autocrine/juxtaparacrine regulation of axon fasciculation by Slit-Robo signaling. Nat. Neurosci. 15, 367–369. 10.1038/nn.3037 22306607

[B30] Jaworski A. Tom I. Tong R. K. Gildea H. K. Koch A. W. Gonzalez L. C.. (2015). Operational redundancy in axon guidance through the multifunctional receptor Robo3 and its ligand NELL2. Science 350, 961–965. 10.1126/science.aad2615 26586761

[B31] Livne I. Gibson M. J. Silverman J. A. (1993). Biochemical differentiation and intercellular interactions of migratory gonadotropin-releasing hormone (GnRH) cells in the mouse. Dev. Biol. 159, 643–656. 10.1006/dbio.1993.1271 8405686

[B32] Low V. F. Fiorini Z. Fisher L. Jasoni C. L. (2012). Netrin-1 stimulates developing GnRH neurons to extend neurites to the median eminence in a calcium- dependent manner. PLoS One 7:e46999. 10.1371/journal.pone.0046999 23056554 PMC3467286

[B33] Mahajan V. S. Demissie E. Mattoo H. Viswanadham V. Varki A. Morris R.. (2016). Striking immune phenotypes in gene-targeted mice are driven by a copy-number variant originating from a commercially available C57BL/6 strain. Cell Rep. 15, 1901–1909. 10.1016/j.celrep.2016.04.080 27210752 PMC4892502

[B34] Marillat V. Cases O. Nguyen-Ba-Charvetm K. T. Tessier-Lavigne M. Sotelo C. Chedotal A. (2002). Spatiotemporal expression patterns of slit and robo genes in the rat brain. J. Comp. Neurol. 442, 130–155. 10.1002/cne.10068 11754167

[B35] Marillat V. Sabatier C. Failli V. Matsunaga E. Sotelo C. Tessier-Lavigne M.. (2004). The slit receptor Rig-1/Robo3 controls midline crossing by hindbrain precerebellar neurons and axons. Neuron 43, 69–79. 10.1016/j.neuron.2004.06.018 15233918

[B36] Matsuo H. Baba Y. Nair R. M. Arimura A. Schally V. A. (1971). Structure of the porcine LH- and FSH-releasing hormone. I. The proposed amino acid sequence. Biochem. Biophys. Res. Commun. 43, 1334–1339. 10.1016/s0006-291x(71)80019-0 4936338

[B37] Messina A. Ferraris N. Wray S. Cagnoni G. Donohue D. E. Casoni F.. (2011). Dysregulation of Semaphorin7A/β1-integrin signaling leads to defective GnRH-1 cell migration, abnormal gonadal development and altered fertility. Hum. Mol. Genet. 20, 4759–4774. 10.1093/hmg/ddr403 21903667 PMC3221532

[B38] Montagutelli X. (2000). Effect of the genetic background on the phenotype of mouse mutations. J. Am. Soc. Nephrol. 11, S101–S105. Available online at: https://jasn.asnjournals.org/content/11/suppl_2/S101.long 11065339

[B39] Nguyen Ba-Charvet K. T. Brose K. Marillat V. Kidd T. Goodman C. S. Tessier-Lavigne M.. (1999). Slit2-Mediated chemorepulsion and collapse of developing forebrain axons. Neuron 22, 463–473. 10.1016/s0896-6273(00)80702-3 10197527

[B40] Nguyen-Ba-Charvet K. T. Brose K. Marillat V. Sotelo C. Tessier-Lavigne M. Chedotal A. (2001). Sensory axon response to substrate-bound Slit2 is modulated by laminin and cyclic GMP. Mol. Cell. Neurosci. 17, 1048–1058. 10.1006/mcne.2001.0994 11414793

[B41] Nguyen-Ba-Charvet K. T. Di Meglio T. Fouquet C. Chédotal A. (2008). Robos and slits control the pathfinding and targeting of mouse olfactory sensory axons. J. Neurosci. 28, 4244–4249. 10.1523/jneurosci.5671-07.2008 18417704 PMC6670299

[B42] Nguyen-Ba-Charvet K. T. Picard-Riera N. Tessier-Lavigne M. Baron-Van Evercooren Sotelo A. C. Chédotal A. (2004). Multiple roles for slits in the control of cell migration in the rostral migratory stream. J. Neurosci. 24, 1497–1506. 10.1523/jneurosci.4729-03.2004 14960623 PMC6730320

[B43] Nguyen-Ba-Charvet K. T. Plump A. S. Tessier-Lavigne M. Chedotal A. (2002). Slit1 and slit2 proteins control the development of the lateral olfactory tract. J. Neurosci. 22, 5473–5480. 10.1523/jneurosci.22-13-05473.2002 12097499 PMC6758232

[B44] Pingault V. Bodereau V. Baral V. Marcos S. Watanabe Y. Chaoui A.. (2013). Loss-of-function mutations in SOX10 cause kallmann syndrome with deafness. Am. J. Hum. Genet. 92, 707–724. 10.1016/j.ajhg.2013.03.024 23643381 PMC3644631

[B45] Pitteloud N. Durrani S. Raivio T. Sykiotis P. G. (2010). Complex genetics in idiopathic hypogonadotropic hypogonadism. Front. Horm. Res. 39, 142–153. 10.1159/000312700 20389092

[B46] Pitteloud N. Quinton R. Pearce S. Raivio T. Acierno J. Dwyer A.. (2007). Digenic mutations account for variable phenotypes in idiopathic hypogonadotropic hypogonadism. J. Clin. Invest. 117, 457–463. 10.1172/jci29884 17235395 PMC1765517

[B47] Plump A. S. Erskine L. Sabatier C. Brose K. Epstein C. J. Goodman C. S.. (2002). Slit1 and Slit2 cooperate to prevent premature midline crossing of retinal axons in the mouse visual system. Neuron 33, 219–232. 10.1016/s0896-6273(01)00586-4 11804570

[B48] Reed K. L. MacIntyre J. K. Tobet S. A. Trudeau V. L. MacEachern L. Rubin B. S.. (2002). The spatial relationship of γ-aminobutyric acid (GABA) neurons and gonadotropin-releasing hormone (GnRH) neurons in larval and adult sea lamprey, Petromyzon marinus. Brain Behav. Evol. 60, 1–12. 10.1159/000064117 12239467

[B49] Sabatier C. Plump A. S. Le M. Brose K. Tamada A. Murakami F.. (2004). The divergent Robo family protein rig-1/Robo3 is a negative regulator of slit responsiveness required for midline crossing by commissural axons. Cell 117, 157–169. 10.1016/s0092-8674(04)00303-4 15084255

[B50] Schwanzel-Fukuda M. Pfaff W. D. (1989). Origin of luteinizing hormone-releasing hormone neurons. Nature 338, 161–164. 10.1038/338161a0 2645530

[B51] Schwarting G. A. Henion T. R. Nugent J. D. Caplan B. Tobet S. (2006). Stromal cell-derived factor-1 (chemokine C-X-C motif ligand 12) and chemokine C-X-C motif receptor 4 are required for migration of gonadotropin-releasing hormone neurons to the forebrain. J. Neurosci. 26, 6834–6840. 10.1523/JNEUROSCI.1728-06.2006 16793890 PMC6673820

[B52] Schwarting G. A. Raitcheva D. Bless E. P. Ackerman S. L. Tobet S. (2004). Netrin 1-mediated chemoattraction regulates the migratory pathway of LHRH neurons. Eur. J. Neurosci. 19, 11–20. 10.1111/j.1460-9568.2004.03094.x 14750959

[B53] Schwarting G. A. Wierman M. E. Tobet A. S. (2007). Gonadotropin-releasing hormone neuronal migration. Semin. Reprod. Med. 25, 305–312. 10.1055/s-2007-984736 17710726

[B54] Sisay S. Pryce G. Jackson S. J. Tanner C. Ross R. A. Michael G. J.. (2013). Genetic background can result in a marked or minimal effect of gene knockout (GPR55 and CB2 receptor) in experimental autoimmune encephalomyelitis models of multiple sclerosis. PLoS One 8:e76907. 10.1371/journal.pone.0076907 24130809 PMC3793915

[B55] Stamou M. I. Georgopoulos A. N. (2018). Kallmann syndrome: phenotype and genotype of hypogonadotropic hypogonadism. Metabolism 86, 124–134. 10.1016/j.metabol.2017.10.012 29108899 PMC5934335

[B56] Sykiotis G. P. Plummer L. Hughes V. A. Au M. Durrani S. Nayak-Young S.. (2010). Oligogenic basis of isolated gonadotropin-releasing hormone deficiency. Proc. Natl. Acad. Sci. U S A 107, 15140–15144. 10.1073/pnas.1009622107 20696889 PMC2930591

[B57] Taroc E. Z. M. Prasad A. Lin J. M. Forni E. P. (2017). The terminal nerve plays a prominent role in GnRH-1 neuronal migration independent from proper olfactory and vomeronasal connections to the olfactory bulbs. Biol. Open 6, 1552–1568. 10.1242/bio.029074 28970231 PMC5665474

[B58] Toba Y. Tiong J. D. Ma Q. Wray S. (2008). CXCR4/SDF-1 system modulates development of GnRH-1 neurons and the olfactory system. Dev. Neurobiol. 68, 487–503. 10.1002/dneu.20594 18188864

[B59] Trarbach E. B. Abreu A. P. Silveira L. F. Garmes H. M. Baptista M. T. Teles M. G.. (2010). Nonsense mutations in FGF8 gene causing different degrees of human gonadotropin-releasing deficiency. J. Clin. Endocrinol. Metab. 95, 3491–3496. 10.1210/jc.2010-0176 20463092 PMC3213864

[B60] Wierman M. E. Kiseljak-Vassiliades K. Tobet S. (2011). Gonadotropin-releasing hormone (GnRH) neuron migration: initiation, maintenance and cessation as critical steps to ensure normal reproductive function. Front. Neuroendocrinol. 32, 43–52. 10.1016/j.yfrne.2010.07.005 20650288 PMC3008544

[B61] Wray S. Grant P. Gainer H. (1989a). Evidence that cells expressing luteinizing hormone-releasing hormone mRNA in the mouse are derived from progenitor cells in the olfactory placode. Proc. Natl. Acad. Sci. U S A 86, 8132–8136. 10.1073/pnas.86.20.8132 2682637 PMC298229

[B62] Wray S. Nieburgs A. Elkabes S. (1989b). Spatiotemporal cell expression of luteinizing hormone-releasing hormone in the prenatal mouse: evidence for an embryonic origin in the olfactory placode. Dev. Brain Res. 46, 309–318. 10.1016/0165-3806(89)90295-2 2655994

[B63] Zelina P. Blockus H. Zagar Y. Peres A. Friocourt F. Wu Z.. (2014). Signaling switch of the axon guidance receptor Robo3 during vertebrate evolution. Neuron 84, 1258–1272. 10.1016/j.neuron.2014.11.004 25433640

[B64] Zhang W. Johnson J. I. Tsai S. P. (2015). Fgf8-deficient mice compensate for reduced gnrh neuronal population and exhibit normal testicular function. Front. Endocrinol. 6:151. 10.3389/fendo.2015.00151 26441841 PMC4585285

[B65] Zhang W. Korstanje R. Thaisz J. Staedtler F. Harttman N. Xu L.. (2012). Genome-wide association mapping of quantitative traits in outbred mice. G3 2, 167–174. 10.1534/g3.111.001792 22384395 PMC3284324

[B66] Zhou S. S. Li P. (2014). Effects of NELL2 on the regulation of GnRH expression and puberty in female rats. Genet. Mol. Res. 13, 6672–6682. 10.4238/2014.august.28.12 25177948

