# Corrigendum: GnRH-1 Neural Migration From the Nose to the Brain Is Independent From Slit2, Robo3 and NELL2 Signaling

**DOI:** 10.3389/fncel.2019.00146

**Published:** 2019-04-24

**Authors:** Ed Zandro M. Taroc, Jennifer M. Lin, Alastair J. Tulloch, Alexander Jaworski, Paolo E. Forni

**Affiliations:** ^1^Department of Biological Sciences, University at Albany, Albany, NY, United States; ^2^Department of Neuroscience, Brown University, Providence, RI, United States

**Keywords:** GnRH-1, olfactory bulbs, Kallmann syndrome, hypogonadotropic hypogonadism, neuronal migration, Robo, Slit, NELL2

In the original article, we neglected to include the funder “National Institutes of Health, R01 NS095908” to AJ and “T32 NS062443 and T32 MH020068” to AT.

The authors apologize for this error and state that this does not change the scientific conclusions of the article in any way. The original article has been updated.

